# Association of hypoalbuminemia and timely albumin infusion with clinical outcomes in patients with sepsis: a retrospective cohort study

**DOI:** 10.3389/fmed.2026.1920038

**Published:** 2026-08-12

**Authors:** Lei Dai, Haoran Zhang, Tongxiang Han, Nana He, Peng Xie, Bin Wang

**Affiliations:** 1 Department of Emergency Internal Medicine, Qingdao Chengyang People's Hospital, Qingdao, Shandong, China; 2 Department of Spinal Surgery, Peking University People's Hospital, Peking University, Beijing, China; 3 Department of Orthopedic Surgery, Weiyuan People's Hospital, Dingxi, Gansu, China; 4 Department of Orthopedic Surgery, Qingdao Chengyang People's Hospital, Qingdao, Shandong, China

**Keywords:** albumin, hospital stay, hypoalbuminemia, mortality, sepsis

## Abstract

**Background:**

Hypoalbuminemia is frequently observed in patients with sepsis. However, its prognostic significance and the association between the timing of albumin administration and clinical outcomes remain uncertain. This study aimed to investigate the association of hypoalbuminemia and timely albumin infusion with clinical outcomes in patients with sepsis.

**Methods:**

This retrospective cohort study included adult patients with sepsis admitted to the hospital. Baseline serum albumin was defined as the lowest albumin level measured within 24 h after admission or sepsis diagnosis. Hypoalbuminemia was defined as serum albumin < 30 g/L. Among patients with hypoalbuminemia, timely albumin infusion was defined as documented albumin administration initiated within 24 h after the first serum albumin level < 30 g/L. The available data captured only the presence and timing of administration, not albumin concentration, dose, duration, or cumulative exposure. The primary outcome was in-hospital mortality. Multivariable logistic regression was used to assess the association between hypoalbuminemia and mortality and, separately, the exploratory association between timely albumin infusion and mortality.

**Results:**

A total of 628 patients with sepsis were included, of whom 296 had hypoalbuminemia and 332 did not. Patients with hypoalbuminemia had higher disease severity scores, higher lactate levels, and a greater need for mechanical ventilation, vasoactive drugs, renal replacement therapy, and intensive care unit admission. Compared with the non-hypoalbuminemia group, the hypoalbuminemia group had significantly higher in-hospital mortality (30.1% vs. 17.8%, *P* < 0.001) and 28-day mortality (35.8% vs. 21.1%, *P* < 0.001). After adjustment for confounding factors, hypoalbuminemia remained associated with increased in-hospital mortality (OR = 1.78, 95% CI: 1.18–2.68, *P* = 0.006). Among patients with hypoalbuminemia, those who received timely albumin infusion had lower in-hospital mortality than those who received delayed or no albumin infusion (22.6% vs. 39.8%, *P* = 0.002). In an exploratory adjusted analysis, timely albumin infusion was associated with lower in-hospital mortality (OR = 0.52, 95% CI: 0.31–0.87, *P* = 0.013); this estimate should not be interpreted as an independent treatment effect.

**Conclusions:**

Hypoalbuminemia was associated with greater disease severity and worse clinical outcomes in patients with sepsis, supporting its role as a prognostic marker. The observed association between timely albumin infusion and lower mortality was exploratory and may be partly explained by residual confounding, treatment-selection bias, and immortal time or survival bias. These findings do not establish a survival benefit or support a therapeutic recommendation for albumin infusion. Prospective studies with detailed time-stamped exposure data and standardized treatment information are required.

## Introduction

Sepsis is a life-threatening clinical syndrome caused by a dysregulated host response to infection and is frequently complicated by acute organ dysfunction. Despite improvements in antimicrobial therapy, fluid resuscitation, hemodynamic monitoring, and organ support, sepsis remains a major cause of death among hospitalized and critically ill patients. Because the clinical course of sepsis can progress rapidly, early recognition of high-risk patients and timely implementation of appropriate interventions are essential for improving prognosis (1, 2).

Hypoalbuminemia is commonly observed in patients with sepsis. Serum albumin is traditionally considered an indicator of nutritional status; however, in acute inflammatory diseases, it also reflects systemic inflammation, endothelial dysfunction, capillary leakage, hepatic synthetic capacity, and disease severity (3). During sepsis, inflammatory cytokine release, increased vascular permeability, redistribution of albumin into the interstitial space, and enhanced protein catabolism may jointly contribute to decreased serum albumin levels. Therefore, hypoalbuminemia may not only represent a laboratory abnormality, but may also serve as a clinically accessible marker of disease burden and poor prognosis (4, 5).

Several studies have reported that reduced serum albumin levels are associated with adverse outcomes in patients with sepsis, including increased mortality, prolonged hospitalization, and a higher requirement for organ support (6, 7). Albumin also has physiological functions relevant to sepsis, including maintenance of plasma oncotic pressure, intravascular volume support, antioxidant activity, endothelial protection, and modulation of inflammatory responses. These properties provide a biological rationale for studying albumin administration. However, observational associations between albumin infusion and outcomes cannot establish treatment efficacy, and the effects may depend on dose, formulation, timing, and clinical context.

Previous studies have mainly focused on baseline albumin levels or albumin infusion in specific populations, such as older intensive care unit patients or patients included in large public databases. Evidence from real-world hospital cohorts remains limited. Patients with sepsis are heterogeneous with respect to infection source, disease severity, comorbidities, nutritional status, timing of diagnosis, and treatment strategies. Therefore, the timing of albumin administration may be associated with outcomes, but such an association requires cautious interpretation because treatment timing is influenced by clinical condition and patient survival.

Therefore, this retrospective cohort study used real-world clinical data to investigate the association between hypoalbuminemia and prognosis in patients with sepsis (8). We also explored whether the timing of albumin infusion was associated with clinical outcomes among patients with hypoalbuminemia. The primary contribution was to evaluate serum albumin as an early prognostic marker; the albumin infusion analysis was considered hypothesis-generating and was not intended to estimate treatment efficacy.

## Methods

### Study design

This was a retrospective cohort study conducted using clinical data from patients with sepsis admitted to our hospital between June 2015 and June 2025. Clinical information was obtained from the hospital electronic medical record system, including demographic characteristics, admission records, laboratory test results, vital signs, comorbidities, infection-related information, treatment measures, and clinical outcomes. This study was performed in accordance with the Declaration of Helsinki and was approved by the Ethics Committee.

### Study population

Patients diagnosed with sepsis during hospitalization were screened for eligibility. Sepsis was defined according to the Sepsis-3 criteria, namely suspected or confirmed infection accompanied by acute organ dysfunction. Adult patients aged 18 years or older were included if they met the diagnostic criteria for sepsis and had at least one serum albumin measurement within 24 h after admission or sepsis diagnosis.

The exclusion criteria were as follows: age younger than 18 years; missing serum albumin data within the early observation window; incomplete key clinical outcome data; hospital stay shorter than 24 h; pregnancy; and patients with end-stage malignant disease or palliative treatment only at admission.

### Definition of hypoalbuminemia

Serum albumin levels were collected from laboratory results within 24 h after admission or sepsis diagnosis. When multiple albumin measurements were available during this period, the lowest value was used as the baseline albumin level. Hypoalbuminemia was defined as serum albumin < 30 g/L. According to baseline serum albumin levels, patients were divided into the hypoalbuminemia group and the non-hypoalbuminemia group.

### Definition of timely albumin infusion

Among patients with hypoalbuminemia, albumin infusion was identified from treatment records. Timely albumin infusion was defined as documented albumin administration initiated within 24 h after the first serum albumin level < 30 g/L. Patients who first received albumin after 24 h or did not receive albumin during hospitalization were classified in the delayed or no albumin infusion group. The 24 h threshold was selected *a priori* as a clinically practical exposure window after recognition of hypoalbuminemia. The available dataset recorded only whether albumin was administered and the timing category; detailed information on albumin concentration or formulation, dose, infusion duration, repeated administrations, and cumulative exposure was unavailable. Accordingly, this analysis evaluated the presence and timing of albumin administration rather than a standardized albumin treatment strategy.

### Data collection

The following variables were collected: age, sex, body mass index, source of infection, comorbidities, laboratory indicators, severity of illness, and major treatment measures. Comorbidities included hypertension, diabetes mellitus, coronary heart disease, heart failure, chronic kidney disease, chronic liver disease, and malignant tumor.

Laboratory indicators included white blood cell count, hemoglobin, platelet count, lactate, serum albumin, total bilirubin, blood urea nitrogen, and serum creatinine. Disease severity was assessed using the Sequential Organ Failure Assessment (SOFA) score and Acute Physiology and Chronic Health Evaluation II (APACHE II) score, depending on data availability. Treatment-related variables included albumin infusion, vasoactive drug use, mechanical ventilation, renal replacement therapy, and intensive care unit admission.

### Outcomes

The primary outcome was in-hospital mortality. Secondary outcomes included 28-day mortality and length of hospital stay. For patients discharged before 28 days, 28-day survival status was obtained from medical records or follow-up records when available.

### Statistical analysis

Continuous variables were presented as mean with standard deviation or median with interquartile range. Categorical variables were presented as numbers and percentages. Differences between groups were compared using the *t*-test or Mann-Whitney U test for continuous variables and the chi-square test for categorical variables.

First, baseline characteristics and clinical outcomes were compared between patients with and without hypoalbuminemia. Second, among patients with hypoalbuminemia, outcomes were compared between the timely albumin infusion group and the delayed or no albumin infusion group. Univariable logistic regression was used to describe associations with in-hospital mortality. Candidate adjustment variables were identified *a priori* on the basis of clinical relevance, previous evidence, and plausible confounding pathways; no automated stepwise selection based solely on univariable *P* values was performed.

The final adjustment set for the primary mortality model included age, sex, body mass index, infection source, diabetes mellitus, chronic kidney disease, chronic liver disease, malignancy, SOFA score, lactate level, serum creatinine, septic shock, and calendar year. All variables in this final set were retained in the adjusted model. Coronary heart disease and heart failure were considered as candidate cardiovascular covariates but were not retained as separate terms to limit overparameterization and clinical overlap with hemodynamic and disease-severity variables. Hypoalbuminemia and serum albumin modeled per 5 g/L decrease were evaluated in separate models and were not entered simultaneously.

Multicollinearity among retained covariates was assessed using variance inflation factors. A variance inflation factor greater than 5 was considered indicative of potentially relevant multicollinearity. Model calibration was evaluated using the Hosmer-Lemeshow goodness-of-fit test and calibration assessment.

Multivariable logistic regression was used to evaluate the adjusted association between hypoalbuminemia and in-hospital mortality. Among patients with hypoalbuminemia, the association between timely albumin infusion and mortality was examined using nested exploratory models: Model 1 adjusted for age and sex; Model 2 additionally adjusted for body mass index, infection source, diabetes mellitus, chronic kidney disease, chronic liver disease, malignancy, and calendar year; and Model 3 additionally adjusted for SOFA score, lactate level, serum creatinine, and septic shock. Because exposure status was assigned using a fixed 24 h window and sufficiently detailed time-stamped data were unavailable, the albumin infusion odds ratios were interpreted as exploratory associations rather than causal or independent estimates of treatment benefit. Results were reported as odds ratio (OR) with 95% confidence interval (CI).

All statistical analyses were performed using SPSS version 27 software. A two-sided *P* value < 0.05 was considered statistically significant.

## Results

### Study population

During the study period, a total of 846 patients with suspected or confirmed sepsis were screened from the electronic medical record system. After excluding patients younger than 18 years old, those with repeated admissions, hospital stay shorter than 24 h, missing early serum albumin data, incomplete outcome information, or palliative treatment only at admission, 628 eligible patients were finally included in the analysis. According to the serum albumin level within 24 h after admission or sepsis diagnosis, 296 patients were assigned to the hypoalbuminemia group and 332 patients were assigned to the non-hypoalbuminemia group. Among patients with hypoalbuminemia, 168 received timely albumin infusion, whereas 128 received delayed or no albumin infusion.

### Baseline characteristics of patients with and without hypoalbuminemia

The baseline characteristics of the included patients are shown in Table 1. The median age of the total cohort was 68.0 years, and 372 patients were male. Compared with patients in the non-hypoalbuminemia group, patients in the hypoalbuminemia group were older and had a higher proportion of septic shock, mechanical ventilation, vasoactive drug use, and intensive care unit admission. Patients with hypoalbuminemia also had higher disease severity scores, including SOFA and APACHE II scores.

**Table 1:** Baseline characteristics of patients with and without hypoalbuminemia.

Variables	Hypoalbuminemia group (*n* = 296)	Non-hypoalbuminemia group (*n* = 332)	*P* value
Age, years	71.0 (63.0–79.0)	66.0 (58.0–75.0)	< 0.001
Male sex, *n* (%)	169 (57.1)	203 (61.1)	0.303
Body mass index, kg/m^2^	22.4 (20.1–25.3)	23.1 (20.8–25.9)	0.046
Hypertension, *n* (%)	132 (44.6)	138 (41.6)	0.444
Diabetes mellitus, *n* (%)	79 (26.7)	82 (24.7)	0.569
Coronary heart disease, *n* (%)	61 (20.6)	58 (17.5)	0.316
Heart failure, *n* (%)	58 (19.6)	43 (13.0)	0.024
Chronic kidney disease, *n* (%)	45 (15.2)	32 (9.6)	0.033
Chronic liver disease, *n* (%)	35 (11.8)	19 (5.7)	0.006
Malignant tumor, *n* (%)	49 (16.6)	39 (11.7)	0.078
Pulmonary infection, *n* (%)	138 (46.6)	137 (41.3)	0.180
Abdominal infection, *n* (%)	71 (24.0)	64 (19.3)	0.151
Urinary tract infection, *n* (%)	38 (12.8)	47 (14.2)	0.624
Bloodstream infection, *n* (%)	26 (8.8)	25 (7.5)	0.564
SOFA score	6.0 (4.0–9.0)	4.0 (2.0–7.0)	< 0.001
APACHE II score	18.0 (13.0–24.0)	14.0 (10.0–20.0)	< 0.001
Septic shock, *n* (%)	104 (35.1)	73 (22.0)	< 0.001
ICU admission, *n* (%)	176 (59.5)	151 (45.5)	< 0.001
Mechanical ventilation, *n* (%)	112 (37.8)	87 (26.2)	0.002
Vasoactive drug use, *n* (%)	121 (40.9)	86 (25.9)	< 0.001
Renal replacement therapy, *n* (%)	39 (13.2)	23 (6.9)	0.008
Lactate, mmol/L	2.8 (1.7–4.6)	1.9 (1.2–3.2)	< 0.001
White blood cell count, × 10^9^/L	13.9 (8.7–20.1)	12.4 (7.9–18.3)	0.087
Hemoglobin, g/L	102.0 (87.0–118.0)	114.0 (98.0–130.0)	< 0.001
Platelet count, × 10^9^/L	151.0 (92.0–236.0)	178.0 (119.0–254.0)	0.004
Serum creatinine, μmol/L	128.0 (82.0–217.0)	102.0 (71.0–168.0)	< 0.001
Blood urea nitrogen, mmol/L	10.6 (6.8–17.4)	7.9 (5.1–13.6)	< 0.001
Total bilirubin, μmol/L	23.0 (12.0–48.0)	18.0 (10.0–34.0)	0.006

SOFA, Sequential Organ Failure Assessment; APACHE II, Acute Physiology and Chronic Health Evaluation II; ICU, intensive care unit.

In terms of laboratory indicators, patients in the hypoalbuminemia group had significantly higher levels of lactate, serum creatinine, blood urea nitrogen, and total bilirubin, whereas hemoglobin and platelet counts were lower than those in the non-hypoalbuminemia group. No significant differences were observed between the two groups in sex distribution, hypertension, diabetes mellitus, or coronary heart disease.

### Clinical outcomes according to hypoalbuminemia status

Clinical outcomes between the hypoalbuminemia and non-hypoalbuminemia groups are summarized in Table 2. Patients with hypoalbuminemia had significantly higher in-hospital mortality than those without hypoalbuminemia (30.1% vs. 17.8%, *P* < 0.001). The 28-day mortality was also higher in the hypoalbuminemia group than in the non-hypoalbuminemia group (35.8% vs. 21.1%, *P* < 0.001).

**Table 2:** Clinical outcomes according to hypoalbuminemia status.

Outcomes	Hypoalbuminemia group (*n* = 296)	Non-hypoalbuminemia group (*n* = 332)	*P* value
In-hospital mortality, *n* (%)	89 (30.1)	59 (17.8)	< 0.001
28-day mortality, *n* (%)	106 (35.8)	70 (21.1)	< 0.001
Length of hospital stay, days	15.0 (9.0–25.0)	12.0 (7.0–20.0)	0.003
Length of ICU stay, days^*^	6.0 (3.0–11.0)	5.0 (2.0–9.0)	0.071

^*^Length of ICU stay was calculated only among patients admitted to the ICU.

In addition, the length of hospital stay was longer in patients with hypoalbuminemia than in those without hypoalbuminemia. Among patients admitted to the ICU, the length of ICU stay was also longer in the hypoalbuminemia group, although the difference was not statistically significant.

### Baseline characteristics of patients according to albumin infusion

Among the 296 patients with hypoalbuminemia, 168 received albumin infusion within 24 h after the first documented serum albumin level below 30 g/L, whereas 128 received albumin after 24 h or did not receive albumin. The baseline characteristics of these two groups are shown in Table 3.

**Table 3:** Baseline characteristics of hypoalbuminemic patients according to timely albumin infusion.

Variables	Timely albumin infusion group (*n* = 168)	Delayed or no albumin infusion group (*n* = 128)	*P* value
Age, years	70.0 (62.0–78.0)	72.0 (64.0–80.0)	0.186
Male sex, *n* (%)	98 (58.3)	71 (55.5)	0.623
Body mass index, kg/m^2^	22.5 (20.2–25.1)	22.2 (19.9–25.5)	0.672
Hypertension, *n* (%)	76 (45.2)	56 (43.8)	0.804
Diabetes mellitus, *n* (%)	44 (26.2)	35 (27.3)	0.829
Chronic kidney disease, *n* (%)	27 (16.1)	18 (14.1)	0.633
Chronic liver disease, *n* (%)	22 (13.1)	13 (10.2)	0.438
Malignant tumor, *n* (%)	30 (17.9)	19 (14.8)	0.485
Pulmonary infection, *n* (%)	77 (45.8)	61 (47.7)	0.752
Abdominal infection, *n* (%)	43 (25.6)	28 (21.9)	0.457
SOFA score	6.0 (4.0–9.0)	6.0 (4.0–8.0)	0.391
APACHE II score	19.0 (14.0–25.0)	17.0 (12.0–23.0)	0.068
Septic shock, *n* (%)	66 (39.3)	38 (29.7)	0.087
Mechanical ventilation, *n* (%)	68 (40.5)	44 (34.4)	0.285
Vasoactive drug use, *n* (%)	76 (45.2)	45 (35.2)	0.081
Renal replacement therapy, *n* (%)	24 (14.3)	15 (11.7)	0.517
Serum albumin, g/L	24.9 (22.1-27.5)	26.7 (23.5-28.9)	0.002
Lactate, mmol/L	3.0 (1.8-4.9)	2.5 (1.5-4.1)	0.041
Serum creatinine, μmol/L	132.0 (86.0-225.0)	121.0 (80.0-206.0)	0.284

Timely albumin infusion was defined as documented albumin administration initiated within 24 h after the first serum albumin level < 30 g/L. The exposure definition reflected timing and presence of administration only and did not represent a standardized dose or treatment protocol.

Patients in the timely albumin infusion group had a slightly higher proportion of septic shock and vasoactive drug use, suggesting that clinicians may have been more likely to administer albumin early to patients with greater circulatory instability. This group also had lower baseline serum albumin levels (24.9 g/L vs. 26.7 g/L, *P* = 0.002) and higher lactate levels (3.0 mmol/L vs. 2.5 mmol/L, *P* = 0.041) than the delayed or no albumin infusion group. There were no significant differences between the groups in age, sex, major comorbidities, infection source, SOFA score, APACHE II score, or serum creatinine.

### Clinical outcomes according to albumin infusion

The clinical outcomes of patients with hypoalbuminemia according to the timing of albumin infusion are presented in Table 4. Compared with patients who received albumin after 24 h or did not receive albumin, those classified as receiving timely albumin infusion had lower in-hospital mortality (22.6% vs. 39.8%, *P* = 0.002) and lower 28-day mortality (29.2% vs. 44.5%, *P* = 0.007).

**Table 4:** Clinical outcomes of hypoalbuminemic patients according to albumin infusion.

Outcomes	Timely albumin infusion group (*n* = 168)	Delayed or no albumin infusion group (*n* = 128)	*P* value
In-hospital mortality, *n* (%)	38 (22.6)	51 (39.8)	0.002
28-day mortality, *n* (%)	49 (29.2)	57 (44.5)	0.007
Length of hospital stay, days	13.0 (8.0–22.0)	17.0 (10.0–28.0)	0.004
Length of ICU stay, days^*^	5.0 (3.0–10.0)	7.0 (4.0–12.0)	0.038

^*^Length of ICU stay was calculated only among patients admitted to the ICU.

The timely albumin infusion group also had shorter observed hospital and ICU stays. These unadjusted comparisons describe associations between exposure groups and should not be interpreted as evidence that early albumin administration caused improved outcomes.

### Multivariable logistic regression analysis for in-hospital mortality

Univariate logistic regression analysis showed that age, SOFA score, lactate level, serum creatinine, septic shock, and hypoalbuminemia were associated with in-hospital mortality.

The multivariable logistic regression models for in-hospital mortality were constructed using the final adjustment set specified in the Methods. All retained covariates were included without automated stepwise selection. Hypoalbuminemia and continuous serum albumin were assessed in separate models. No significant multicollinearity was observed; variance inflation factors for all retained covariates were below 5, with a maximum value of 2.31. The model showed acceptable calibration according to the Hosmer-Lemeshow goodness-of-fit test (*P* = 0.642).

After adjustment for the specified covariates, hypoalbuminemia remained associated with higher odds of in-hospital mortality in patients with sepsis (adjusted OR = 1.78, 95% CI: 1.18–2.68, *P* = 0.006). In a separate model in which serum albumin was analyzed as a continuous exposure, each 5 g/L decrease was associated with higher odds of in-hospital mortality (adjusted OR = 1.31, 95% CI: 1.12–1.54, *P* = 0.001) (Table 5).

**Table 5:** Multivariable logistic regression analysis for in-hospital mortality in patients with sepsis.

Variables	Unadjusted OR (95% CI)	*P* value	Adjusted OR (95% CI)	*P* value
Age, per 10-year increase	1.28 (1.09–1.51)	0.003	1.19 (1.01–1.42)	0.041
Male sex	1.08 (0.74–1.58)	0.684	1.03 (0.68–1.55)	0.894
SOFA score, per 1-point increase	1.24 (1.17–1.32)	< 0.001	1.16 (1.08–1.25)	< 0.001
Lactate, per 1 mmol/L increase	1.18 (1.10–1.27)	< 0.001	1.11 (1.03–1.21)	0.009
Serum creatinine, per 50 μmol/L increase	1.12 (1.05–1.20)	0.001	1.06 (0.98–1.15)	0.147
Septic shock	2.68 (1.84–3.91)	< 0.001	1.71 (1.08–2.70)	0.022
Hypoalbuminemia	1.99 (1.37–2.89)	< 0.001	1.78 (1.18–2.68)	0.006
Serum albumin, per 5 g/L decrease	1.46 (1.27–1.68)	< 0.001	1.31 (1.12–1.54)	0.001
Body mass index, kg/m^2^	0.95 (0.90–1.01)	0.094	0.97 (0.91–1.04)	0.382
Pulmonary infection	Reference		Reference	
Abdominal infection	1.34 (0.86–2.08)	0.192	1.22 (0.74–2.02)	0.432
Urinary tract infection	0.86 (0.46–1.60)	0.632	0.91 (0.46–1.81)	0.786
Bloodstream infection	1.76 (0.89–3.47)	0.103	1.48 (0.69–3.16)	0.312
Diabetes mellitus	1.12 (0.74–1.70)	0.594	1.03 (0.64–1.66)	0.904
Chronic kidney disease	1.69 (1.02–2.79)	0.041	1.23 (0.68–2.23)	0.491
Chronic liver disease	1.87 (1.04–3.37)	0.037	1.42 (0.73–2.78)	0.303
Malignant tumor	1.78 (1.08–2.94)	0.024	1.51 (0.86–2.66)	0.153
Calendar year, per 1-year increase	0.97 (0.92–1.03)	0.342	0.98 (0.92–1.04)	0.514

SOFA, Sequential Organ Failure Assessment. The adjusted models included age, sex, body mass index, infection source, diabetes mellitus, chronic kidney disease, chronic liver disease, malignant tumor, SOFA score, lactate level, serum creatinine, septic shock, and calendar year. Hypoalbuminemia and serum albumin per 5 g/L decrease were evaluated in separate models and were not entered simultaneously.

Among patients with hypoalbuminemia, timely albumin infusion remained associated with lower in-hospital mortality in the fully adjusted exploratory model (adjusted OR = 0.52, 95% CI: 0.31–0.87, *P* = 0.013) (Table 6). Because the exposure was classified using a fixed 24 h window, this adjusted OR may be influenced by immortal time or survival bias and should not be interpreted as an independent estimate of treatment benefit.

**Table 6:** Association between timely albumin infusion and in-hospital mortality among patients with hypoalbuminemia.

Analysis model	OR (95% CI)	*P* value
Unadjusted model	0.44 (0.27–0.72)	0.001
Model 1: adjusted for age and sex	0.46 (0.28–0.76)	0.002
Model 2: additionally adjusted for body mass index, infection source, diabetes mellitus, chronic kidney disease, chronic liver disease, malignant tumor, and calendar year	0.49 (0.29–0.82)	0.007
Model 3: additionally adjusted for SOFA score, lactate, serum creatinine, and septic shock	0.52 (0.31–0.87)	0.013

SOFA, Sequential Organ Failure Assessment. Timely albumin infusion was defined as documented albumin administration initiated within 24 h after the first serum albumin level < 30 g/L. The reported OR represent exploratory associations and not causal treatment effects.

## Discussion

In this retrospective hospital-based cohort, hypoalbuminemia was common among patients with sepsis and was associated with greater disease severity, greater use of organ support, and worse clinical outcomes. After multivariable adjustment, hypoalbuminemia remained associated with higher in-hospital mortality, supporting serum albumin as a prognostic marker. Among patients with hypoalbuminemia, timely albumin infusion was associated with lower mortality in an exploratory analysis. However, this latter finding cannot establish a treatment benefit and may be partly explained by residual confounding, treatment-selection bias, and survival bias arising from the fixed exposure window. Thus, the principal contribution of this study is the prognostic information provided by early hypoalbuminemia, whereas the albumin infusion analysis should be viewed as hypothesis-generating.

Hypoalbuminemia is frequently observed in patients with sepsis and critical illness. Traditionally, serum albumin has been regarded as a marker of nutritional status; however, in the setting of acute infection and systemic inflammation, hypoalbuminemia reflects a more complex pathophysiological process. During sepsis, inflammatory mediators can suppress hepatic albumin synthesis, increase vascular endothelial permeability, promote leakage of albumin into the interstitial space, and accelerate protein catabolism. In addition, fluid resuscitation, hemodilution, renal dysfunction, hepatic impairment, and gastrointestinal protein loss may further contribute to decreased serum albumin levels. Therefore, serum albumin is not only a nutritional indicator, but also a comprehensive marker of inflammatory burden, endothelial injury, circulatory instability, and disease severity (9–11).

Recent evidence further supports the prognostic importance of albumin-based composite biomarkers in patients with sepsis (12). Duran et al. investigated the prognostic value of the red blood cell distribution width-to-albumin ratio (RAR) in patients with sepsis and reported that higher RAR values were associated with increased mortality. Their study suggested that RAR had better prognostic performance than red blood cell distribution width or albumin alone, highlighting the clinical utility of combining inflammatory and nutritional indicators in risk stratification. This finding is consistent with our observation that hypoalbuminemia was independently associated with increased in-hospital mortality in patients with sepsis. However, our study differs from that work in several aspects. While Duran et al. focused on RAR as a prognostic biomarker in sepsis patients, the present study primarily evaluated serum albumin itself and further explored the association between timely albumin infusion and clinical outcomes among septic patients with hypoalbuminemia. Taken together, these findings suggest that albumin has prognostic value both as an individual laboratory parameter and as a component of ratio-based biomarkers reflecting inflammation, hematologic stress, and nutritional-metabolic status.

In the present study, patients with hypoalbuminemia had higher SOFA and APACHE II scores, higher lactate levels, and a greater requirement for mechanical ventilation, vasoactive drugs, and renal replacement therapy. These findings indicate that hypoalbuminemia was closely associated with more severe organ dysfunction and poorer physiological reserve. The increased mortality observed in patients with hypoalbuminemia may be explained by several mechanisms (13–17). First, decreased albumin levels reduce plasma colloid oncotic pressure, which may aggravate intravascular volume depletion, tissue edema, and microcirculatory dysfunction. Second, hypoalbuminemia may impair the transport of endogenous and exogenous substances, including hormones, fatty acids, drugs, and inflammatory mediators. Third, albumin has antioxidant and anti-inflammatory properties, and reduced albumin levels may weaken the ability to counteract oxidative stress and excessive inflammation. Fourth, hypoalbuminemia may be a marker of persistent catabolism and poor nutritional reserve, both of which are associated with impaired immune function and delayed recovery from infection.

Our findings are consistent with previous studies showing that low serum albumin is associated with poor prognosis in sepsis. Importantly, serum albumin as a prognostic marker and albumin infusion as a therapeutic exposure represent distinct clinical questions. The present data support the former more directly: early hypoalbuminemia identified patients with greater organ dysfunction and higher mortality. By contrast, the infusion analysis cannot determine therapeutic efficacy. Although patients classified as receiving timely albumin infusion had lower mortality and the association persisted after covariate adjustment, exposure assignment required patients to remain alive and under observation long enough to receive albumin within the 24 h window. This creates the possibility of immortal time or survival bias. Because sufficiently detailed time-stamped data were unavailable, time-dependent modeling or a landmark analysis could not be performed. The adjusted OR should therefore be interpreted only as an exploratory association, not as an independent estimate of treatment benefit.

Albumin has several physiological properties that provide a plausible rationale for future interventional research. It contributes to plasma oncotic pressure and intravascular volume, binds endogenous and exogenous molecules, and may exert antioxidant and endothelial effects (18–21). Nevertheless, biological plausibility does not validate the observed association in this retrospective analysis. The clinical effect of albumin may vary substantially according to concentration or formulation, dose, duration, cumulative exposure, fluid status, hemodynamic context, and concurrent treatments, none of which could be adequately characterized in the present dataset.

The clinical implications should therefore be framed cautiously. Early serum albumin measurement may assist risk stratification because hypoalbuminemia identifies septic patients with greater disease burden and a higher likelihood of adverse outcomes. It may also prompt a broader assessment of inflammation, capillary leakage, hepatic function, nutritional status, fluid balance, and organ dysfunction. However, the present findings do not support recommending albumin infusion solely to correct hypoalbuminemia or on the basis of the 24 h exposure definition used in this study. Decisions regarding albumin administration should remain guided by the overall clinical context and established sepsis management principles. Whether a standardized early albumin strategy improves survival requires prospective evaluation.

The strengths of this study include the use of real-world hospital data and the simultaneous evaluation of two related but distinct questions: the prognostic significance of hypoalbuminemia and the exploratory association between albumin infusion timing and outcomes. The analysis adjusted for multiple clinically relevant covariates and clearly identified early hypoalbuminemia as a marker of increased risk. The infusion analysis adds a hypothesis for future research but should not be considered evidence of therapeutic effectiveness.

Several limitations should be acknowledged. First, this was a retrospective observational study, and causal relationships cannot be established. Residual confounding and treatment-selection bias are likely because albumin administration was determined by clinicians and may have reflected disease severity, hemodynamic status, fluid balance, or other undocumented factors. Second, immortal time bias is central to the albumin infusion analysis. Patients classified as receiving albumin within 24 h necessarily had to survive and remain eligible long enough to receive it, whereas early deaths could preferentially enter the delayed or no albumin group. This survival bias may partly or even substantially explain the observed association. Exact time-stamped data were insufficient for time-dependent exposure modeling or landmark analysis; therefore, the adjusted OR cannot be interpreted as a causal or independent treatment effect. Third, the dataset did not contain reliable details on albumin concentration or formulation, dose, infusion duration, repeat administration, or cumulative exposure. The study therefore evaluated only the presence and timing of administration, not a standardized treatment strategy. Fourth, unmeasured factors, including liver disease severity, nutritional assessment, frailty, capillary leakage, code status, cumulative fluid balance, hemodynamic trajectories, and concurrent nutritional or fluid therapies, may have influenced the results. Fifth, hospital discharge may act as a competing event for analyses of hospital mortality and length of stay. These limitations reinforce that the infusion findings are exploratory and require prospective confirmation.

## Conclusions

In conclusion, early hypoalbuminemia was associated with greater disease severity and worse clinical outcomes in patients with sepsis, supporting its use as a prognostic marker. Among patients with hypoalbuminemia, timely albumin infusion was associated with lower in-hospital mortality in an exploratory analysis; however, this association may be affected by residual confounding, treatment-selection bias, and immortal time or survival bias. It should not be interpreted as evidence that albumin replacement improves survival or as a basis for therapeutic recommendations.

Prospective multicenter studies, ideally with detailed time-stamped exposure data and standardized information on albumin formulation, dose, duration, and cumulative exposure, are needed to determine whether any albumin treatment strategy improves outcomes and to identify the patients most likely to benefit.

## Data Availability

The raw data supporting the conclusions of this article will be made available by the authors, without undue reservation.
